# Draft Genome Sequence of Streptococcus parasuis 4253, the First Available for the Species

**DOI:** 10.1128/MRA.00203-19

**Published:** 2019-05-02

**Authors:** Marc J. A. Stevens, Nicole Cernela, Sabrina Corti, Roger Stephan

**Affiliations:** a Institute for Food Safety and Hygiene, Vetsuisse Faculty, University of Zurich, Zurich, Switzerland; University of Maryland School of Medicine

## Abstract

Here, we report the draft genome sequence of Streptococcus parasuis strain 4253. This is the first publicly available genome sequence of a *S. parasuis* strain.

## ANNOUNCEMENT

Streptococcus parasuis is a species that consists of members that formerly belonged to Streptococcus suis serotypes 20, 22, and 26 (1). It is closely related to S. suis but can be distinguished from it based on genetic content and biochemical features, such as the absence of both β-galactosidase activity and arginine hydrolyses (1, 2). Strains can easily be classified as *S. parasuis* based on a positive PCR targeting a 679-bp fragment near the *recN* gene (2).

*S. parasuis* has been isolated almost exclusively from saliva of both healthy and diseased pigs. Until now it was unclear whether *S. parasuis* is a pathogen, like its close relative S. suis, or whether it is a member of the commensal microbiota of pigs (2).

*S. parasuis* 4253 was isolated from a tube of a teat sealer which was applied to a dried-up cow suffering from subsequently lethal mastitis. The teat sealer (1 g) was enriched at 37°C for 24 h under aerobic conditions in brain heart infusion broth (Thermo Fisher Diagnostics AG, Pratteln, Switzerland) and thereafter streaked on Columbia blood sheep agar (Thermo Fisher Diagnostics AG) and incubated for 48 h at 37°C under aerobic conditions.

Genomic DNA was isolated with the DNA blood and tissue kit (Qiagen, Hombrechtikon, Switzerland). The DNA was sequenced with a Nextera DNA Flex sample preparation kit (Illumina, San Diego, CA, USA) and a MiniSeq sequencer (Illumina). The sequencing output was 1,018,561 150-bp paired-end reads. Reads were checked for quality with the software package FastQC 0.11.7 (Babraham Bioinformatics, Cambridge, UK) and were assembled with SPAdes 3.12 and Shovill 1.0.4, a tool that accelerates SPAdes (3, 4), with default settings. The assembly was filtered, retaining contigs larger than 500 bp and with coverage more than 25-fold. The draft genome sequence of *S. parasuis* 4253 consists of 1,881,656 bp divided over 106 contigs with an *N*_50_ length of 25,681 bp and a largest contig size of 125 kbp. The GC content of the genome is 39.9 mol%, very close to the 39.8 mol% recorded for the type strain SUT-286^T^ (1). The genome encodes 1,867 protein-coding genes and 39 tRNAs. The number of rRNA operons was estimated to be four.

An *in silico* PCR tool (5) using our genome as the template and *S. parasuis*-specific primers resulted in an expected 679-bp fragment, thereby confirming the species identification of strain 4253 as *S. parasuis*.

The genome sequence of *S. parasuis* 4253 might provide information about the spread of this bacterium in different hosts and environments. This is the first available genome sequence of a strain identified as *S. parasuis*.

### Data availability.

This whole-genome shotgun project has been deposited at DDBJ/ENA/GenBank under the accession number SHGT00000000. The version described in this paper is version number SHGT01000000. Reads were deposited in the Sequence Read Archive under accession number SRX5381921.
